# The many faces of p53: something for everyone

**DOI:** 10.1093/jmcb/mjz026

**Published:** 2019-08-15

**Authors:** Arnold J Levine

**Affiliations:** Institute for Advanced Study, Princeton, NJ 08540, USA

## The p53 gene, from discovery to classification: the first 10 years

Forty years ago four research laboratories in London, Paris, New York/Bethesda, and Princeton uncovered the existence of the p53 protein (Deleo et al., 1979; Lane and Crawford, 1979; Linzer and Levine, 1979; Kress et al., 1979). Each laboratory came upon this protein for a different reason and with a different experimental approach that uncovered this unanticipated result. Together, the four papers permitted one to conclude the following: (i) in SV40-infected and transformed cells the SV40-encoded oncogene protein, the large T-antigen, formed a protein complex with a cellular-encoded protein of ~53000 daltons in size. (ii) This p53 protein was detected at high levels in a variety of transformed cells derived from viral, chemical, or inherited (teratocarcinomas) transformation events. (iii) Nontransformed cells expressed lower levels of the p53 protein. (iv) Animals bearing tumors produced antibodies directed against the p53 protein.

A temperature-sensitive mutation in the SV40 large T-antigen gene (the oncogene of this virus) was employed to demonstrate that the p53–T-antigen complex was formed at the permissive temperature, where the cells are transformed, but not at the nonpermissive temperature, where the cells behave normally (Linzer and Levine, 1979; Linzer et al., 1979). At a later date p53 protein complexes with viral oncogene products were observed, including the adenovirus E1b-58kd protein (Sarnow et al., 1982) and the human papilloma virus E6 oncoprotein (Scheffner, et al. 1990; Werness et al., 1990; which is the cause of human cervical cancers and some head and neck cancers).

In order to explore the functions of the p53 protein, several p53 cDNAs were isolated and cloned (Oren and Levine, 1983; Oren et al., 1983; Pennica et al., 1984). These clones were tested for oncogene activities and found to cooperate with the RAS oncogene in transforming embryonic cells (Eliyahu et al., 1984; Parada et al., 1984). Thus, it appeared that the p53 gene was an oncogene whose protein forms a complex with viral oncogene proteins, possibly mediating transformation. However, the cDNA clone isolated by Pennica failed to transform cells in culture and had a single amino acid change when compared with the Oren cDNA clone, which did transform cells. Was the amino acid difference between these clones significant? Was this difference a sequencing mistake? A polymorphism? Or a mutation? If it was a mutation, which clone was the wild-type and which was the mutant? To address these questions, Oren and Levine exchanged clones (and reproduced each other’s observations). By 1989 it became clear that mutations in the p53 cDNA clones resulted in cellular transformation, and wild-type p53 protein prevented transformation and functioned as a tumor suppressor (Eliyahu et al., 1989; Finlay et al., 1989). p53 mutations in both p53 alleles in colon cancers of humans resulted in the same conclusion; p53 functioned as a tumor suppressor gene that helped to prevent cancer (Baker et al., 1990a, b, Nigro et al., 1995).

From 1979 to 1989 the p53 protein was alternatively referred to as a fetal antigen expressed in the teratocarcinoma stem cells, a tumor antigen that induced antibodies in animals and humans with tumors, an oncogene whose mutant forms could transform cells, and, finally, a tumor suppressor gene that prevented cancers. During this time the p53 protein was demonstrated to increase its concentration in response to DNA damage (Maltzman and Czyzyk, 1984). Over these first 10 years of research the p53 protein was shown to have many diverse faces and activities, functioning as an oncogene and a tumor suppressor gene while responding to DNA damage in a cell.

## The mutants of the p53 gene: an extraordinary diversity

The mutant forms of p53 add to the many faces of p53. There are multiple ways to inactivate p53 protein functions. There are mutations such as deletions, nonsense mutations, and frameshift mutations, which are all true loss-of-function mutations, but more commonly there are missense mutations localized in the p53 protein DNA-binding domain (Bouaoun et al., 2016). In addition, there are gene amplifications in the *MDM-2* gene that overexpress this ubiquitin ligase, which is a negative regulator of the levels of the p53 protein. Thirdly, there are protein modifications that reduce or eliminate p53 transcriptional activity (Zhu et al., 2016; Levine, 2017). To date, >500 different missense mutations, located in the DNA-binding domain of the p53 protein, have been isolated and sequenced from human cancers. Missense mutations that produce high levels of mutant p53 proteins can function as dominant negative proteins, inactivating the wild-type p53 protein, in cells with normal levels of wild-type proteins. This is why mutant p53 cDNA clones transform normal cells in culture. A tetramer of the wild-type p53 protein functions as the transcription factor. Faulty or mutant monomeric proteins contributing to the tetrameric protein poison its activity and act in a dominant negative fashion. A second way in which missense mutant p53 proteins act in cells is to contribute a gain of function to the cell. This is observed when a missense mutant protein is added to a cell with no p53 protein (a deletion mutation). The cell clone expressing the missense protein is then compared to the cell clone with no p53 protein. When this is done the cells expressing a mutant missense p53 protein have new activities that enhance transformation and tumorigenesis (Dittmer et al., 1993). Thus, the mutant protein, by itself, has gained new functions.

The 500 plus different missense mutations in the p53 gene that have been observed in cancers occur at different frequencies in all types of human cancers from 7% for some alleles to 0.005% for other mutant alleles. The top 10 mutant alleles account for 33% of human cancer mutations, whereas the top 50 mutant alleles account for ~50% of human cancer mutations. Thus, the frequencies with which different mutant p53 alleles are observed in many human cancers occur over a four log difference in their levels (Balachandran et al., 2017; Levine, 2019). Why? These 500 mutant proteins have a wide variety of structures. There are both mutations that alter amino acids that make a contact with the template DNA sequence, and there are mutations that alter the structure of the p53 DNA-binding domain. Different missense mutants transcribe seven different p53-responsive genes in a yeast cell assay at a wide variety of different efficiencies (Kato et al., 2003; Mathe et al., 2006; Petitjean et al., 2007). The top 10 most frequent mutant p53 alleles largely fail to transcribe p53-responsive genes. Some p53 mutant alleles produce proteins that can act to induce a CD-8 T-cell response in long-term survivors of cancers (Łuksza et al., 2017; Levine, 2018, 2019). The immunological activity of a p53 mutation depends upon the binding efficiency of HLA-A, HLA-B, or HLA-C class 1 receptor alleles and the presence of a T-cell receptor that recognizes the mutant peptide, giving rise to an extraordinary diversity of the immune response to p53 mutant proteins in human populations.

The extraordinary diversity of mutant missense p53 alleles, occurring at different frequencies with different phenotypes, and the diversity of the immune system in response to p53 mutant alleles have led Sabapathy and Lane (2018) to term this ‘a rainbow of p53 mutations’ where ‘some mutations are more equal than others’. Does this mean that it will take a rainbow of drugs to treat cancers with p53 mutations (Levine, 2019)?

## The multiplicity of functions of the p53 protein

The p53 protein is a transcription factor. It binds to a degenerate DNA sequence (Pu,Pu,Pu,C,A/T,A/T,G, Py,Py,Py) repeated twice with a variable spacer, where Pu is a purine, Py a pyrimidine, and A, T, G, and C are the four usual bases. About 200–300 genes are thought to be regulated at the transcription level by the p53 protein, but this is at best an estimate. Indeed, a mega-analysis of several publications exploring p53-regulated genes has identified only ~60 genes found in common in all the publications. The p53 gene enhances the transcription of the MDM-2 gene, which produces the ubiquitin ligase that promotes the degradation of the p53 protein. This produces an autoregulatory loop so that p53 and MDM-2 proteins oscillate out of phase (Wu et al., 1993). Thus, p53 protein levels are regulated at the posttranslational level (Oren et al., 1982). The half-life of the p53 protein is quite short, between 6 and 20 min. When a cell is exposed to one or many stresses, termed the input signals, posttranslational modifications of the MDM-2 and p53 proteins increase the half-life of the p53 protein and activate it for transcription. Table 1 lists some of the p53 input signals. It is striking that a wide variety of diverse stresses activate the transcriptional activity of the p53 protein. The activated p53 protein responds to different stresses by expressing a number of transcriptional programs, which are output signals of p53-mediated transcription that are listed in Table 2. These outputs may result in quite different outcomes, resulting in cell death or the repair of the consequences of stressful inputs. There are two p53 transactivation domains, and they regulate different sets of genes as outputs (Lin et al., 1994; Raj and Attardi, 2016). In addition to the posttranslational modifications of p53, the oscillations, the alterations in these oscillations, or the lack of oscillations all can influence the output transcriptional programs (Stewart-Ornstein et al., 2017). The output transcriptional program mediated by an activated p53 protein can be influenced by the cell type, a stem or progenitor cell, or a differentiated cell, whether or not the cell is transformed or normal, or even by the nature of the microbiome (bacterial or viral).

**Table 1:** p53 input signals.

**Stress signal**		**Mediator**	**Act upon**
1.	DNA damage	ATM, ATR, ChK-1 and ChK-2	MDM-2; p53 ↑
2.	Telomere erosion	ATM, ChK-2	MDM-2; p53 ↑
3.	Metabolism	Starvation, XTPs	MDM-2; p53 ↑
4.	Ribosomal biogenesis	Ribosomal proteins	MDM-2; p53 ↑
5.	Hypoxia, anoxia	HIF-1α, DNA damage	p53 ↑
6.	Oncogene activation	Alternative Reading Frame (ARF)	MDM-2; p53 ↑
7.	Redox potential	ROS, glutathione	p53-cyst-277
8.	Mitophagy of cytokines that make ROS	Pink, Parkin	Mitochondria
9.	Epigenetic changes	Histone, acetylation, methylation, etc.	MDM-2; p53 ↑
10.	Infectious diseases, viruses, papilloma;	E6	p53 ↓
	bacteria, helicobacter	CAG-A-AKT increases MDM-2	p53 ↓
		iASP–p53 complex	p53 ↓
11.	Inflammation	NF-κB, cytokines	p53↓
12.	Cortisol stress	SGK-1 modifies MDM-2	p53↓
13.	Aging	?	p53↓ with age

XTPs, nucleoside triphosphates; ARF, alternative reading frame; ROS, reactive oxygen species.

**Table 2:** p53 output signals.

**Impact**		**Regulated by**	**Result**
1.	Cell cycle arrest	p21, 14-3-3-σ, CDC-25A, Aurora-A, GADD-45, mir-34	G-1 and G-2 arrest
2.	Apoptosis	Puma, Noxa, Bax, APAF-1, p53 AIP-1, TNF, FASL, mir-34c	Death
3.	Senescence	mir-34a, PML, PAI-1, p21, secretory cytokines	Permanent arrest; cytokines
4.	DNA repair	p53 R2, Ercc-5, FANC-C, XPC, Ku86, Gadd-45a	Repair damage
5.	Metabolism	GLS2, T1GFR, PTEN, TSC-2, ALDH-4, P1G-3, SCO2, mir-34a	Warburg to homeostasis
6.	ROS	(a) Sestrin 1, Sestrin 2, GLS-2	ROS inactivation
		(b) PINK-1, Parkin	Mitophagy
7.	ncRNAs	mir-34a	Apoptosis, cell cycle arrest, senescence, metabolic regulation
8.	Pluripotent stem cells	(a) p21	Initiate differentiation
		(b) Methylation of p53-inactive ↓	Enhance stem cell division
9.	Negative regulators	MDM-4, MDM-2, Wip-1, iASP, methyltransferases	p53↓
10.	Positive regulators	ASPP-1, ASPP-2, PML	p53 ↑
11.	Epigenetic regulation	Many activities of protein modifiers	p53 ↓, ↑

The input and output signal transduction pathways place p53 and MDM-2 at a central hub that extends out to a very large number of diverse functions and other signal transduction pathways (Vogelstein et al., 2000). It has become possible to identify connections among many different signal transduction pathways that carry out a large number of cellular functions creating a network of cellular interactions. In this network the central p53 hub communicates with a large number of inputs (Table 1) and responds with a large number of outputs (Table 2). The p53 hub both receives and transmits information about the intracellular and extracellular environment. It has become possible to calculate the amount of information (entropy) that each hub in the network receives and transmits. It turns out that the p53 hub has the highest information content when compared to all other nodes in the network of a cell (Pouryahya et al., 2018). It is not surprising that the p53 node in the network has maximal entropy in that it deals with the reproduction of the cell and the efficient extraction of energy from nutrients, two important evolutionary properties. Changes in entropy are positively correlated with a notion of robustness and create an architecture where information is processed through multiple cellular hubs (Tannenbaum et al., 2015). Cellular stresses during replication result in an enhanced rate of mutations or mistakes, and under these conditions p53 ensures fidelity by repair or by death. Surely one of the reasons why the p53 hub has control over an extraordinary level of cellular information is that there are so many diverse stresses that input to the p53 hub. Why is this? It would appear that this configuration, where many different types of stress act through a single gene and protein (p53), to ensure a response, would create a vulnerable node, liable to failure if that gene is nonfunctional via a mutation. Why not build a network with 10 different stress responders for 10 stresses and then connect them together? The most common explanation for the existing configuration (one stress responder) is that two or more different stress signals at the same time must be integrated or communicated to result in a proper response. Networks are better at integrating this information at a single node (protein) than by requiring additional connections among nodes that must coordinate the response. If this is correct, then p53 takes on the informational function of coordinating situations where multiple things go wrong by receiving and then integrating the information before programing the right kind of outputs. All of this is consistent with p53 having a position in the network that ensures a higher order of information transfer integrating the signals. This may well be the reason why the p53 gene is the single most common gene to be mutated in human cancer.

The higher order function of the p53 gene and protein is to ensure fidelity and homeostasis by integrating responses to stresses. Furthermore, this explanation would suggest that no one or two or even three downstream p53-regulated genes are responsible for tumor suppression by the p53 gene. So, deletion of selected downstream genes without loss of tumor suppression is not a surprise. The p53 circuitry is designed to compensate for that possibility. Rather, tumor suppression is a regulated, integrated, and informed coordinated set of responses by the p53 protein to environmental perturbations resulting in the maintenance of cellular homeostasis. It seems likely that the communication of stress signals to p53 is mediated by protein modifications from the epigenetic activities that also communicate with chromatin, histones, and other transcription factors helping to integrate the information about environmental changes. That confers maximum information content upon the p53 node. It may be of some interest that the set of nodes in the cellular network with the second highest information content is the set of 14-3-3 genes that position various proteins at cellular locations, permitting functions or preventing them. With p53 involved in enforcing homeostasis under conditions of varying environments and 14-3-3 proteins creating a regulated topological framework for cellular proteins, these two sets of nodes act through time and space to provide cellular information. The 14-3-3σ gene and protein are regulated in part by the p53 protein (Yang et al., 2003) connecting these high-entropy pathways. The 14-3-3σ protein induced by p53 results in a G-2 cell cycle arrest and inhibits the AKT kinase activity that plays an important role in survival and cell proliferation in some cancers (Yang et al., 2006).

## Where did the p53 gene come from? Why and how did the p53 gene become a central player in multiple cellular functions and take on a central role in cancers?

The amino acid sequence of the human p53 protein DNA-binding domain (amino acids 100–320 out of 393) is conserved over a period of about one billion years of evolution (Belyi and Levine, 2009). The present day examples of placozoans, derived from the oldest evolutionary ancestors of today’s animals, contain an amino acid sequence with a p53-like DNA-binding domain (Lane et al., 2010). These are among the simplest or most primitive multicellular organisms. Moving up the evolutionary tree, sea anemones, flat worms, and fruit flies have been characterized with a p63-like DNA-binding domain, a close relative of p53 in humans. Remarkably, both the DNA-binding domain protein structures and the specific DNA sequence it binds with are conserved in the p53/p63-like molecules from sea anemones to humans (Belyi et al., 2010). In all of these invertebrates there is a single p53-like gene, which is expressed in the germ line tissues but not the somatic tissues. If the germ-line DNA of these organisms is damaged by radiation, then p53 is activated and kills the sperm or egg precursor cells. Death is by apoptosis, and even the genes in the p53-activated apoptotic pathway are conserved from invertebrates to vertebrates. In flat worms, starvation of the parents activates p53 and germ cells are killed. This is remarkably similar to anorexia in human females, where p63 kills germ cells due to starvation (Levine et al., 2011). Thus, the p53 ancestor gene is first observed in primitive multicellular organisms, ensuring fidelity of the germ-line DNA sequences under stress (radiation and starvation) by killing cells with irreparably damaged DNA. Darwinian evolution proceeds by generating enough diversity in a species to permit natural selection to act and to create new species that are better adapted to changes in the environment. The p53-like gene arises to prevent the generation of too much diversity through catastrophic errors that are not repaired or eliminated. It is difficult for an organism to survive and replicate efficiently with too many changes of its information. At the extreme, this is called the error catastrophe threshold, and some viruses, such as influenza A and human immunodeficiency virus, live and reproduce at this extreme. Organisms that are at this extreme can utilize resources but function and reproduce poorly. Thus, the p53/p63 precursors of invertebrates counter too much diversity and ensure fidelity.

A gene with functions like p53 has its origins in the germ line of invertebrates. Many invertebrates undergo development through successive larval forms, and the sexually reproducing adult is largely postmitotic. Only the germ line cells divide and propagate in sexually active adults. With the advent of vertebrate organisms, a new strategy emerges. The sexually reproducing adult develops stem cells that regenerate somatic cells and tissues many times over the lifetime of the organism. With the appearance of tissue-specific stem cells and tissue regeneration, lifetimes of organisms lengthen, and the stem cells of the body accumulate mutations. As a stem cell population acquires mutations, a natural selection for stem cell clones that out-replicate other stem cells in each organism arises. At this point Darwinian evolution is functioning within both somatic stem cell and germ cell lineages of an organism. With additional mutations, cancers develop from these clones and ultimately alter tissue regeneration and functions of the organism. It is at about this time, in cartilaginous and bony fishes, when the single invertebrate p53/p63-like gene expands into three vertebrate genes, p53, p63, and p73, each of which takes on diverse functions. Based upon the rate and extent of amino acid changes in the DNA-binding domain of these three genes, the p53 gene evolves the most rapidly and dramatically, while p63 and p73 genes change only modestly. p53 activities move from the germ line to the somatic tissues, enhancing fidelity and homeostasis in somatic tissue-specific stem and progenitor cells. It becomes a tumor suppressor gene and expands its role as the central stress responder in cells. As the p53 gene evolves from invertebrates to vertebrates it becomes repurposed from the germ line to somatic tissue-specific stem cells. Its functions largely remain the same to ensure fidelity by death. p63 retains its functions of homeostasis and fidelity of the female germ line in vertebrates (Belyi et al., 2010) while becoming a major stem cell factor for skin production. The p73 gene controls flagella formation in several tissues during development and functions in many tissues, including male sperm cell development, the central nervous system, and the immune system (Nemajerova et al., 2018).

## The research focus of the p53 field upon cancers did not have to happen this way—what the future might hold

Mutations in the p53 gene occur in ~50% of human cancers, making it the most commonly altered gene in human cancers. Of the 80000 plus publications about the p53 gene and protein, the great majority of them focus on cancer. p53 meetings are all about cancers. It did not have to happen this way. The reason for a cancer focus in the p53 field was that all four of the discovery papers of the p53 protein were about cancers and viruses that caused cancer, and almost all the investigators who moved into this field were cancer biologists. But the p53 gene and protein function as a stress responder, reviewed in Table 1, and these stresses have an impact upon many tissue and organ functions that can lead to many diverse disorders or can even regulate normal organismic functions (Table 2). It is clear that p53, p63, and p73 play an important role in reproduction (Levine et al., 2011). The p53 gene plays a role in regulating the implantation of fertilized eggs into the uterus and the formation of the placenta. It does so by regulating the transcription of the LIF gene. LIF is required for implantation (Hu et al., 2007, 2009; Feng et al., 2011). The placenta sets up a barrier between the immune system of the mother and the fetus. LIF is one of the hormones that helps mediate that barrier to T-cell rejection. LIF is secreted by a number of tumors (even when p53 is mutated) and antibodies directed against LIF aid in tumor rejection by the immune system. Perhaps the study of placental barriers to the immune system would be instructive in understanding some of the mechanisms that block the immune rejection of the embryo and of tumors *in vivo* in the adult. In addition, tumors employ a number of normal developmental processes to protect themselves, replicate, and metastasize, and we can learn a great deal from understanding the role of p53 in the movement of cells during development, immune tolerance, and autoimmunity.

Tables 1 and 2 make it abundantly clear that the p53 gene is a part of the innate immune system. It is intimately involved with both the microbiome and infectious diseases. Both the DNA and RNA viruses (not only the tumor viruses) induce p53 activity in virus-infected cells. Successful viruses have developed countermeasures to inactivate the p53 activity that reduces the viral replicative functions. A number of intracellular bacteria and bacteria that interact with the cell surface can activate a p53 response, which can kill the infected cell. These activated innate p53 functions signal to attract macrophages and monocytes, which process the antigens of the microbiome for responses by the adaptive immune system (Tanne et al., 2015). Had the p53 protein been discovered by immunologists studying infectious diseases, the focus of the p53 field would have been quite different.

p53-mediated cell senescence, by either oncogene activation or DNA damage, can result in a p53 secretory pathway that calls forth natural killer (NK) cells that kill the senescent cells and macrophages that eliminate the dead cells. Indeed, one of the hypotheses of why cellular, organ, and organismic aging occurs with time is that mutations accumulated with age trigger p53-activated senescent cells that are then eliminated by NK and myeloid cells. As we age the efficiency of the hematopoietic system declines; the cells are not eliminated; and a chronic secretion of inflammatory cytokines result in age-related diseases (arthritis, neurodegenerative diseases, diabetes and metabolic abnormalities, cardiovascular diseases, autoimmune diseases, etc.). With aging, replicative senescence results in shorter telomeres. The p53 response to this is cell cycle arrest. It is not surprising then that high levels of chronic p53 activity in cells can bring about accelerated aging (Kastenhuber and Lowe, 2017). There is an interesting association between aging and alterations in the DNA methylation profiles in somatic cells of the body (Horvath and Raj, 2018). At selected sites in the genome, alterations occur in the levels of methylated cytosine residues at CpG dinucleotides as a function of increasing age. However, there are differences between individuals when chronological age is compared with biological age, which is determined by the rate of methylated CpG alterations with time and not the number of years after your birthday. The available evidence (Feng et al., 2007; Yi et al., 2012, 2014; Levine, 2015, 2017) suggests that p53 plays a role in enforcing the stability and homeostasis of CpG epigenetic changes in cells. This is yet another relationship between aging programs and p53 gene and protein functions. What remains now is to develop a clear understanding of the role of p53 in contributing to the aging program in each organism and diverse species. Each species has a characteristic distribution of the number of years a member of the species will live. Is there a p53 function that differs between species and interacts with other functions that contribute to this age-dependent distribution of life span? Understanding the relationships between p53 pathway functions with developmental changes and time will relate the entropic forces of the p53 network with our programmed lifespan.

The genomes of most organisms are decorated with the ghosts of transposons and retrotransposons that have entered the germ line in the evolutionary past. In many organisms there is a short time period in early development when viable and active transposons can replicate and move around the genome creating polymorphisms and even initiating functional changes such as adding new transcription binding sites to genes adjacent to integration sites and changing regulatory circuits. This seems to favor rapid evolutionary change and diversity. After this the retrotransposons are heavily methylated at CpG residues, and transcription and transposition are shut down for the remainder of life. In some pathological conditions like cancers (with p53 mutations) methylated CpG stability is lost, and these elements are activated and create a genomic instability (Levine et al., 2016). Here again p53 enforces epigenetic homeostasis resulting in a genomic homeostasis and slows diversity of the genome (Levine et al., 2016). These relationships open up a new opportunity for drug development of epigenetic-modifying activities.

Another important player that enforces genomic stability is DNA repair. The many different DNA repair pathways respond to different types of DNA damage. Every free-living organism has evolved and kept DNA repair pathways demonstrating the central feature this plays in survival and reproduction. In humans there are a large number of such pathways and perhaps 300–350 genes dedicated to these functions. It has become clear that a significant number of early onset human cancers have inherited defects in these DNA repair genes. It is also clear that p53 regulates the transcription of a number of genes in different DNA repair pathways (Table 2) and this is part of its normal tumor suppressor functions. It is certainly possible that p53 would be maximally efficient in DNA repair if it transcribed the gene with a rate-limiting step of that repair pathway (and this could be tissue-dependent). Understanding the regulation and the interactions between DNA repair pathways and how they may provide redundant functionality could be useful in designing new approaches not only to cancer treatments but also to many other diseases of the ederly.

Evolution places a high priority for selective forces upon reproduction and efficient energy use obtained from food. A listing of the p53-regulated genes that have an impact upon metabolic functioning (Table 2) demonstrates that p53 affects glucose metabolism, glutamine metabolism, lipid and fatty acid production, glutathione regulation of reactive oxygen, and mitochondrial function. These processes have an impact upon the switch between Warburg metabolic processes with utilization of high glucose levels for rapid growth (rapid cell division in early development, wound repair, and cancer) and optimal energy production (adenosine triphosphate, ATP) from glucose producing carbon dioxide and water in mitochondria (Levine and Puzio-Kuter, 2010). The p53-regulated genes focus upon major substrates (glucose or glutamine) and major pathways used for rapid replication versus homeostatic maintenance of the organism. There is more to learn here.

The above discussion shows that the areas and topics for possible future exploration of the p53 gene and protein have several things in common. They all are biological processes found in free-living organisms from bacteria to humans and have strong evolutionary forces acting to optimize them. From sea anemones to humans, they all have common evolving p53 functions that impact each area of research: (i) reproduction, (ii) metabolism, (iii) genomic and epigenetic stability, (iv) DNA repair, (v) transposon control, (vi) a life span program and aging, and (vii) infectious disease and the immune system. Each of these areas of research can be affected adversely by stress, responds with p53 activity, and downstream (output) genes that either correct the effects of stress or eliminate the cell so that the multicellular organism will not be adversely affected. Evolution appears to have first identified the p53 gene and selected it for protecting reproductive genomic, and possibly epigenomic, stability in early invertebrates. In worms metabolic stress (starvation) was connected to the germ line functions, and so food and sex were probably the early uses of p53 stress responses. In shrimps and clams transposon control was added to p53 functions in somatic tissues to prevent leukemia by transposition and insertional activation. By the fishes and in the higher vertebrates all seven of the functions were being developed and modified. Any one of these biological processes that utilize p53 to integrate responses to stress may well develop into a field with its own set of interests and impacts, and it is likely that topics not yet on this list will find a home for the p53 gene and protein. The many faces and functions of the p53 gene and protein are to be expected because stress, as defined broadly herein, impacts upon the most important biological processes needed for life and successful reproduction. The p53 gene and protein is one example of a long list of genes identified, selected for, and kept by evolution over one billion years. This list ought to be our focus of the study of life and life processes at the molecular level.

*[The manuscript benefited from the editing of Ms Suzanne Christen. This work was supported by a grant from the National Institutes of Health—National Cancer Institute (5PO1CA087497-17). The author is a founder, member of the board of directors, and stockholder of PMV Pharmaceuticals, which develops structural correctors for p53 mutant alleles.]*
